# CD44v6 Targeted by miR-193b-5p in the Coding Region Modulates the Migration and Invasion of Breast Cancer Cells

**DOI:** 10.7150/jca.35067

**Published:** 2020-01-01

**Authors:** Song Hu, Manlin Cao, Yiqing He, Guoliang Zhang, Yiwen Liu, Yan Du, Cuixia Yang, Feng Gao

**Affiliations:** 1 Department of Molecular Biology, Shanghai Jiao Tong University Affiliated Sixth People's Hospital, Shanghai 200233, China.; 2 Department of Rehabilitation Medicine, Shanghai Jiao Tong University Affiliated Sixth People's Hospital, Shanghai 200233, China.; 3 Department of Clinical Laboratory, Shanghai Jiao Tong University Affiliated Sixth People's Hospital, Shanghai 200233, China.

**Keywords:** Breast cancer, miR-193b-5p, migration, invasion, biomarker

## Abstract

Previous studies have shown that CD44 containing variant exon v6 (CD44v6) is highly expressed in many cancers and is related to tumor metastasis. However, the detailed mechanism of the regulatory pattern of CD44v6 in breast cancer remains unclear. Here, we found that CD44v6 was significantly upregulated in invasive breast cancer cell lines compared with low-invasive breast cancer cell lines. Cell migration and invasion could be suppressed by CD44v6 downregulation. MiRWalk and RNAhybrid software revealed miR-193b-5p as a miRNA targeting CD44v6 by binding to the exon v6 region. We found that the overexpression of miR-193b-5p inhibited the migration and invasion of Hs-578t and BT-549 cells, which could be rescued by restoring the expression of CD44v6. Next, we determined the potential of miR-193b-5p as an *in vitro* biomarker for breast cancer. Serum samples were obtained from 58 breast cancer patients, 36 patients with benign disease and 58 age-matched cancer-free controls. The results showed that the expression of miR-193b-5p in the serum was significantly lower in breast cancer patients than in controls and could distinguish cancer from cancer-free samples. The area under the receiver operating characteristic curve (ROC) for miR-193b-5p was 0.762(95% confidence interval: 0.674-0.851), which was higher than that of carcinoembryonic antigen (CEA) and cancer antigen 15-3 (CA15-3). Combining miR-193b-5p with CEA or CA15-3 could improve the diagnostic efficiency compared with the CEA and CA15-3 combination. Taken together, our results suggest that miR-193b-5p could function as a tumor-suppressive miRNA by targeting CD44v6 in breast cancer and that serum miR-193b-5p may serve as a biomarker for breast cancer diagnosis.

## Introduction

Breast cancer is the most common cancer among women, with an annual incidence of 2,088,849 and a mortality of 626,679 worldwide 1. Most of the deaths from breast cancer are due to metastasis to other organs, such as lung 2, bone 3 and brain 4. Despite recent advances in the diagnosis and treatment options for breast cancer, tumor metastasis still leads to low survival rates 5. Therefore, a better understanding of the underlying mechanisms of metastasis is critical for identifying novel biomarkers and for developing new therapies.

CD44 containing variant exon v6 (CD44v6), an important isoform of CD44, is largely involved in various physiological processes. The aberrant expression of CD44v6 has been found in many cancers, such as colorectal cancer 6, ovarian cancer 7 and prostate cancer 8. The role of CD44v6 has been shown to be associated with tumor metastasis. A meta-analysis of 921 patients showed that the high expression of CD44v6 in non-small cell lung cancer tissues was associated with lymph node metastasis and histological type 9. The expression level of CD44v6 and its clinical pathological grade and stage were negatively correlated in urothelial carcinoma of the bladder 10. CD44v6 was also considered a marker of cancer stem cells that drove cancer metastasis in colon cancer 11. Moreover, CD44v6 shows great value in clinical application. High expression levels of CD44v6 were positively correlated with poor overall survival in patients with gastric cancer 12 and epithelial ovarian cancer 13. CD44v6 could acquire drug resistance to 5-fluorouracil and L-OHP in SW480 cells by modulating autophagy 14. Soluble CD44v6 has great potential as a biomarker. The expression of soluble CD44v6 in the serum could be used to discriminate patients from controls in children with B-cell acute lymphoblastic leukemia (B-ALL) 15. In stage I/II colorectal cancer, the diagnosis based on combined soluble CD44v6 and CEA is more valuable than that of CD44v6 or CEA alone 16. Thus, CD44v6 plays an important role in tumorigenesis and metastasis. However, in addition to the proven mechanisms in other tumors, little is known about the regulatory pattern of CD44v6 in breast cancer.

MicroRNAs (miRNAs) are 19-24 nt noncoding RNAs that can bind to their target genes in the 3'-Untranslated Region (3'-UTR) and inhibit translation 17, 18. More recently, it was established that miRNAs can regulate the expression of target genes by binding to their coding area 19, 20. Previous studies have shown that miRNAs could regulate cancer development by targeting CD44. miR-34a-5p could inhibit metastasis and cancer stem cells by directly repressing CD44 in prostate cancer^21^. CD44 was also suggested as a direct target of miR-199a-3p in hepatocellular carcinoma 22 and osteosarcoma 23. Furthermore, expression of the CD44 3'UTR could serve as a competitor for miR-216a, miR-330, and miR-608 binding to regulate tumorigenesis and angiogenesis 24. However, the miRNAs that target CD44v6 in breast cancer are still poorly understood.

In this study, we report that CD44v6 expression in invasive breast cancer cell lines is associated with tumor migration and invasion. We further determined that CD44v6 was targeted by miR-193b-5p and that the miR-193b-5p:CD44v6 axis can regulate the migration and invasion of breast cancer. The expression of miR-193b-5p in serum samples indicated that it may act as a biomarker for diagnosis. Therefore, our findings reveal that miR-193b-5p is a potent inhibitor of tumor migration and invasion by targeting CD44v6 and could serve as a potential biomarker for breast cancer diagnosis.

## Materials and methods

### Patients and specimens

Human breast cancer serum samples and the corresponding control serum samples were obtained from Shanghai Jiao Tong University Affiliated Sixth People's Hospital. A total of 152 serum samples were examined in this study, including 58 samples collected from patients with breast cancer before surgery, 36 samples from benign tumor patients and 58 normal serum samples from age- and sex-matched healthy volunteers. This study was approved by the Research Ethics Committee of Shanghai Jiao Tong University Affiliated Sixth People's Hospital.

### Cell culture and antibodies

Human breast cancer cell lines (MCF-7, T-47D, Hs-578t, BT-549, and HEK293) were purchased from the Cell Bank of the Type Culture Collection of the Chinese Academy of Sciences (Shanghai, China). MCF-7 cells were cultured in MEM(Gibco,USA); T-47D and BT-549 cells were cultured in RPMI-1640 medium(Gibco, USA); and Hs-578t and HEK293 cells were cultured in high-glucose DMEM (Gibco, USA).All cell lines were cultured at 37 °C in humidified air with 5% CO_2_ and 95% air and the media were supplemented with 10% fetal bovine serum and 100 IU/ml penicillin/streptomycin. An anti-CD44v6 mouse monoclonal antibody (Clone #2F10) was purchased from R&D Systems, and an anti-CD44 mouse monoclonal antibody (ab119348) was purchased from Abcam.

### Quantitative real-time PCR (qRT-PCR)

For CD44v6, total RNA was extracted from cultured cells (MCF-7, T-47D, BT-549, and Hs-578t) by RNAiso Plus (Takara, Japan). RNA (1 μg) was reverse transcribed with the PrimeScript™ RT Regent Kit with gDNA Eraser (Takara, Japan). Real-time PCR assays were performed by using SYBR Green Mix (Takara, Japan) according to the manufacturer's protocol. All qRT-PCR values of each gene were normalized against that of GAPDH. The relative expression of genes was calculated by the 2^-ΔΔCt^ method.

For miR-193b-5p, total miRNA from serum samples was extracted using the miRNeasy Serum/Plasma Kit (QIAGEN, German) in accordance with the manufacturer's protocol. cDNA synthesis was performed using the miRcute Plus miRNA First-Strand cDNA Synthesis Kit (TianGen, China) and miRNA amplification was performed using the miRcute Plus miRNA qPCR Detection Kit (TianGen, China). All q-PCR values of each gene were normalized against that of *Caenorhabditis elegans* (cel)-miR-39, which was spiked during the total RNA extraction process. The primer sequences in this study are shown in Table S1.

### Plasmid constructs and RNA oligonucleotides

Human CD44v6 was cloned into the pCMV-Flag vector (Clontech, Mountain View, CA, USA). CD44 exon v6 and mutated CD44v6 were cloned downstream of the Renilla luciferase coding sequences in pRL-TK (Promega). These constructs were confirmed by DNA sequencing. The miR-193b-5p mimic, the control mimic, the siRNA targeting CD44v6 and scramble RNAs were purchased from Ribobio (Guangzhou, China). The primer sequences in this study are shown in Table S1.

### Cell transfection and Western blotting

The miR-193b-5p mimic, pCMV-CD44v6 plasmid and their respective control RNAs were transfected into Hs-578t and BT-549 cells using Lipofectamine 3000 transfection reagent (Invitrogen, USA) according to the manufacturer's protocol. Additionally, 50 nM miRNA and 0.5 μg of pCMV-CD44v6 plasmid were used in a 6-well plate. Protein and RNA were collected 48 h after transfection. RIPA buffer (Beyotime, China) was used for protein extraction. After the total protein concentration was determined by a bicinchoninic acid protein assay kit (Sigma, USA), 30 μg protein samples were separated by 8% SDS polyacrylamide gels and transferred onto PVDF membranes(Millipore, Billerica, USA). The membrane was blocked with 5% nonfat milk in TBST for 1 h and incubated with the indicated antibody (CD44v6, R&D Systems. BBA13, 1:1000; CD44s, CST #5640, 1:1000; GAPDH, 1:5000) at 4 °C overnight. Then HRP-conjugated secondary antibodies (1: 5000) were added. Bands were subsequently visualized using the enhanced plus chemiluminescence assay (Pierce, USA). Measurement of the bands was conducted on an ImageQuant LAS 4000 mini.

### Dual-luciferase reporter assay

HEK293 cells were transfected with 0.5 μg of pRL-TK-CD44v6/Mut, together with 50 nM miR-193b-5p mimic or the cognate control RNA via Lipofectamine 3000(Invitrogen, USA). After 48 h, dual-luciferase reporter assays were performed using the Dual Luciferase Reporter Assay System (Promega) according to the manufacturer's protocol.

### Cell proliferation and plate colony formation

The cell proliferation of Hs-578t and BT-549 cells was measured via CCK-8 assay (KeyGen Biotech,China) according to the manufacturer's protocol. In brief, cells were transfected with miRNAs and/or plasmids. After 24 h, equal numbers of cells (2000 cells/well) were seeded into 96-well plates for the CCK-8 assay. For colony formation, 300 cells/well were plated into 6-well plates and all plates were incubated for 2 weeks to allow colony formation. The cells were fixed with 4% paraformaldehyde for 30 min and stained with 0.1% crystal violet (Beyotime) for 30 min. After rinsing three times, the stained colonies were photographed.

### Cell migration and invasion assays

Cell migration was measured by a wound healing assay. Hs-578T and BT-549 cells were transfected for 24 h and cultured in 6-well plates. Until breast cancer cells formed 100% confluent monolayers, the cultures were scratched by using a 20-μl pipette tip. The medium was replaced with DMEM or RPMI-1640 containing 0.2% FBS, and then the cells were incubated for an additional 24 hours. The migration areas were measured by ImageJ software.

To evaluate the invasion ability, transwell assays were performed. In brief, Hs-578t and BT-549 cells were suspended in medium containing 0.2% FBS after transfection. In addition, 5×10^4^ cells were seeded into the upper chamber of an 8-μm pore size insert with Matrigel (BD Biosciences, USA). The chambers were deposited in a 24-well plate with 600μl of 10% FBS medium. After 24 h, the cells were fixed with 4% paraformaldehyde (Beyotime, China) and stained with crystal violet (Beyotime, China). After removing the cells on the upper surface of the chamber, the penetrated cells were captured at 200×magnification in six random fields under a microscope, and the number of cells was counted by ImageJ software.

### Statistical analysis

All data are presented as the mean±SD and were analyzed with GraphPad Prism 7 and SPSS v23 software. Receiver operating characteristic (ROC) curve analysis was used to evaluate diagnostic performance. Logistic regression was performed to acquire the sensitivity and specificity of the optimum combination of miR-193b-5p, CEA, and CA15-3.Student's t-test was used to identify the differences between the treated groups and their controls. A P value<0.05 was considered statistically significant in the text and figures (*P<0.05, **P<0.01, ***P<0.001).

## Results

### CD44v6 expression is associated with the migration and invasion of breast cancer

**Figure 1:**
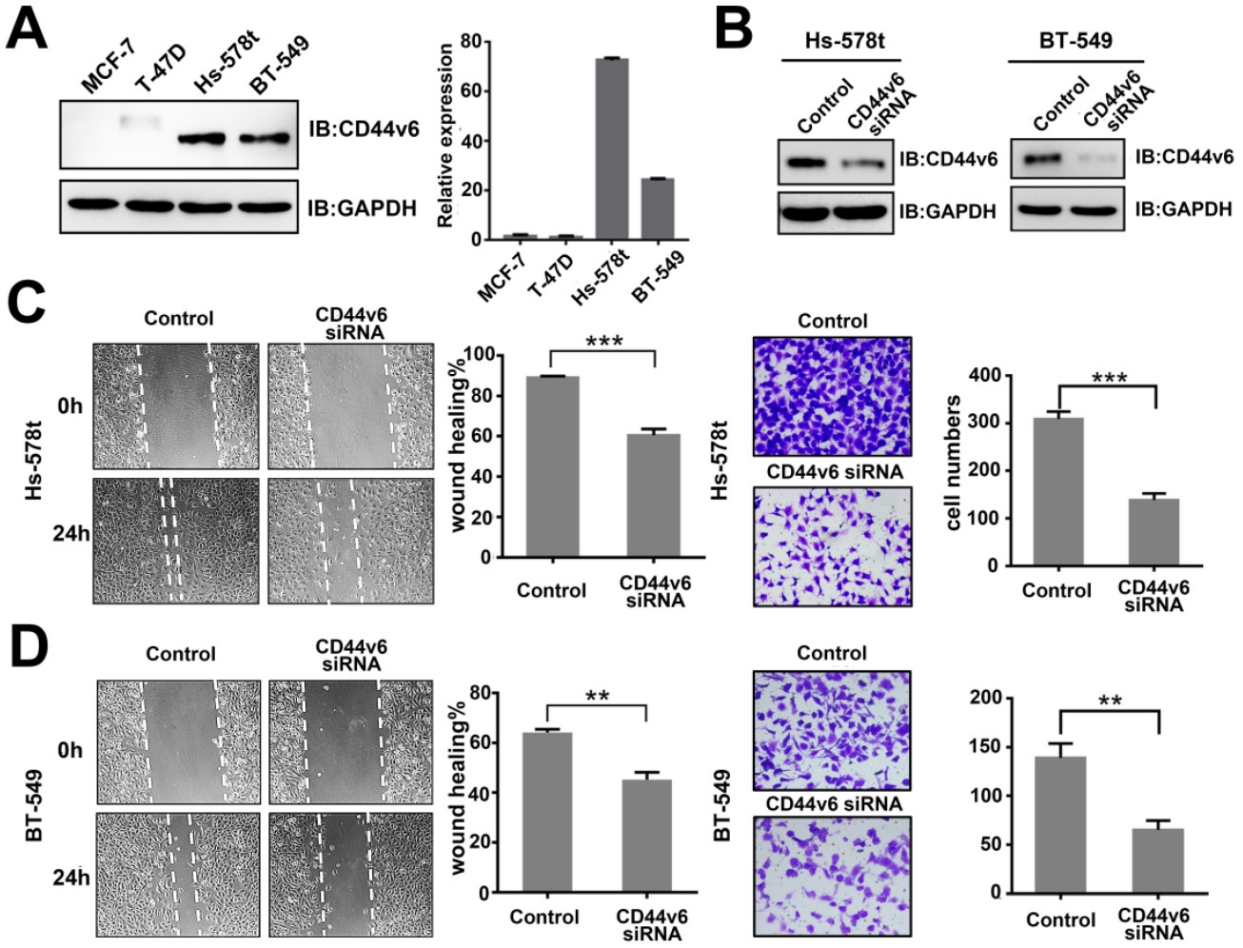
** CD44v6 expression is associated with the migration and invasion of breast cancer.** (A) Expression of CD44v6 in the breast cancer cell lines MCF-7, T-47D, Hs-578t, and BT-549. Left, western blot analysis of CD44v6 protein; Right, qRT-PCR analysis of CD44v6 mRNA levels.(B) Immunoblot analysis of CD44v6 expression in Hs-578t and BT-549 cells transfected with CD44v6 siRNA. (C-D) Migration (left, wound healing) and invasion (right, transwell) assays for control and CD44v6 knockdown Hs-578t (upper panel) and BT-549 (bottom panel) cells. Quantitative results of migratory cells are shown. Data show the mean±SD of three independent experiments. **P<0.01;***P<0.001.

To clarify the function of CD44v6 in breast cancer, we first screened the expression of CD44v6 in a panel of breast cancer cell lines. Immunoblot analysis and qRT-PCR both showed that the expression of CD44v6 was relatively higher in invasive breast cancer cells Hs-578t and BT-549, and lower in MCF-7 and T-47D cells (Figure 1A). This result suggests that CD44v6 is a relatively higher expressed in invasive breast cancer cells than in low-invasive breast cancer cells.

To characterize CD44v6 activity, we suppressed CD44v6 by transfection with specific a siRNA in Hs-578t and BT-549 cells. The expression of CD44v6 in both cell lines was dramatically inhibited (Figure 1B). Then, we performed wound healing and Matrigel cell invasion assays. The invasive capability of Hs-578t and BT-549 cells was dramatically attenuated by CD44v6 siRNA compared with control cells (Figure 1C and D). These data show that CD44v6 repression is associated with metastasis in breast cancer.

### miR-193b-5p represses CD44v6 translation via a regulatory element in exon v6

**Figure 2:**
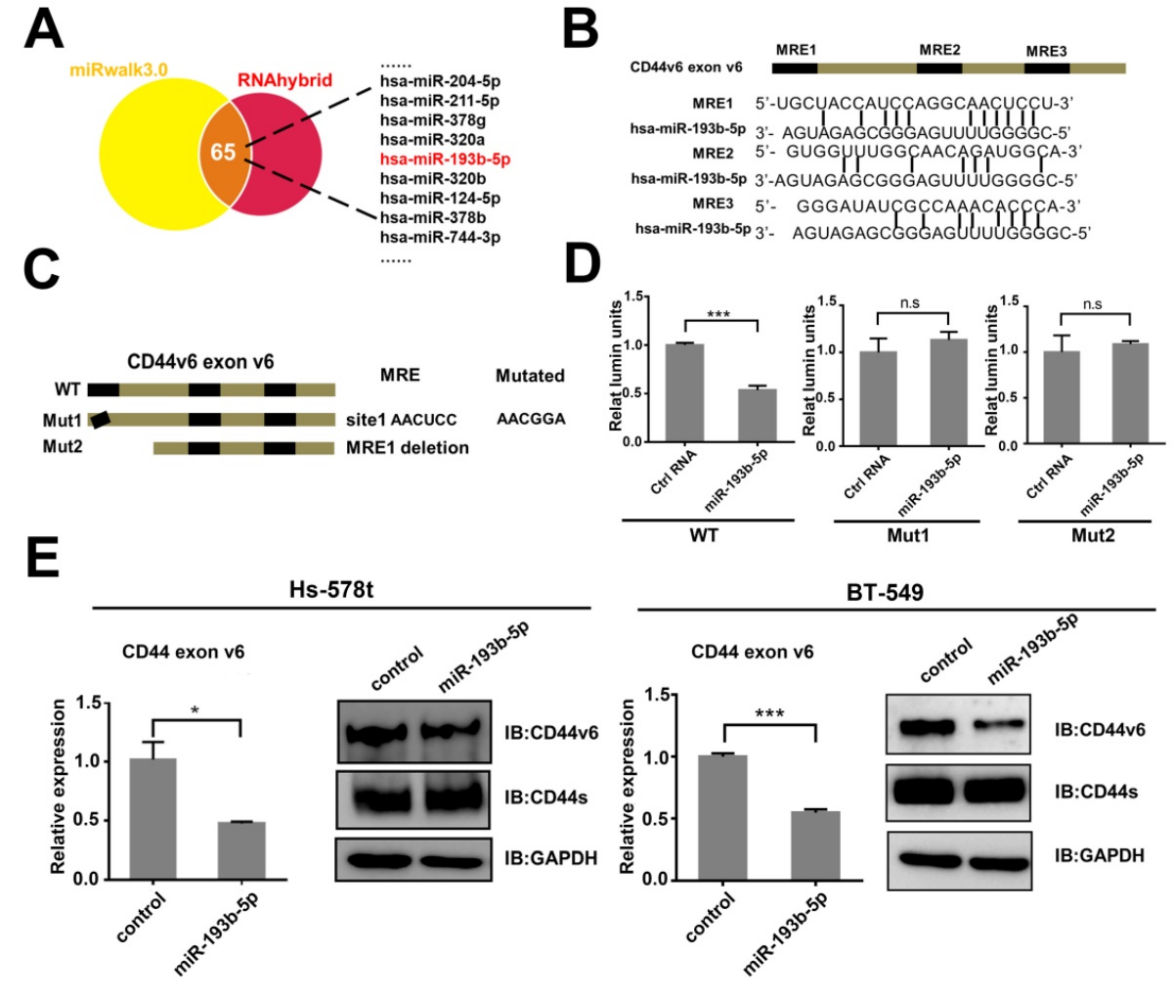
** miR-193b-5p targets CD44v6 via a regulatory element in exon v6** (A) Potential miRNAs targeting CD44v6 predicted by miRNA-target prediction programs (miRwalk and RNAhybrid). (B) Potential MREs in exon v6 of CD44v6 mRNA. Top, schematic representation of three putative MREs in exon 6; bottom, the sequences of four potential MREs. (C) Schematic representation of synonymous mutations (Mut1) and deletion (Mut2) at the putative MREs. (D) Luciferase reporter assays in HEK293 cells, indicating that miR-193b-5p targets CD44v6 in MRE1. (E) Western blot (left) or qRT-PCR analysis of CD44v6 expression (right) in Hs-578t and BT-549 cells transfected with miR-193b-5p mimic, *P<0.05; **P<0.01;***P<0.001, P=ns. ns, no significance.

To our knowledge, the CD44v isoforms and CD44s shared the conserved untranslated region (UTR) 25 and constant exons 26. Then, to explore the specific miRNA binding to CD44v6, we screened the miRNAs that may target CD44v6 in exon v6 with miRwalk software, which integrated results from other databases^27^ and confirmed by RNAhybrid software 28(Figure 2A). Using this method, we identified 65 candidate miRNAs. We transfected eleven miRNAs into BT-549 cells and examined the protein level of CD44v6 by Western blot analysis (Figure S1 A). The results showed that miR-193b-5p, miR-378b, and miR-204 inhibited the expression of CD44v6. qRT-PCR were performed to detect the expression of the three miRNAs in breast cancer cell lines (Figure S1 B). miR-193b-5p was expressed at lower levels in Hs-578t and BT-549 cells than in MCF-7 and T-47D cells. However, the expression of miR-378b and miR-204 was irregular in breast cancer cell lines. These results suggest that miR-193b-5p may target CD44v6.

To test whether miR-193b-5p regulated CD44v6 via this exon, we searched exon v6 and found three putative MREs (microRNA response elements) (Figure 2B). A truncation and synonymous mutations were constructed (Figure 2C). These three sequences were cloned downstream of the Renilla luciferase cDNA in the pRL-TK vector. These luciferase reporter vectors containing the exon v6 sequence or mutated sequences were cotransfected into HEK293 cells with an NC-mimic control or miR-193b-5p-mimic. The luciferase assay results showed that only mutations at MRE1 completely abrogated the repressive effect of miR-193b-5p on CD44v6 expression. This result suggests that miR-193b-5p could directly target CD44v6 (Figure 2D). To further confirm the relationship between miR-193b-5p and CD44v6, the influence of miR-193b-5p overexpression (Figure S1 C) on CD44v6 was explored. The results showed that miR-193b-5p could downregulate the expression of CD44v6 in Hs-578t and BT-549 cells. These data indicate that miR-193b-5p represses CD44v6 at the posttranscriptional level by binding to elements in exon v6.

### miR-193b-5p inhibits the migration and invasion of breast cancer cells triggered by CD44v6

**Figure 3:**
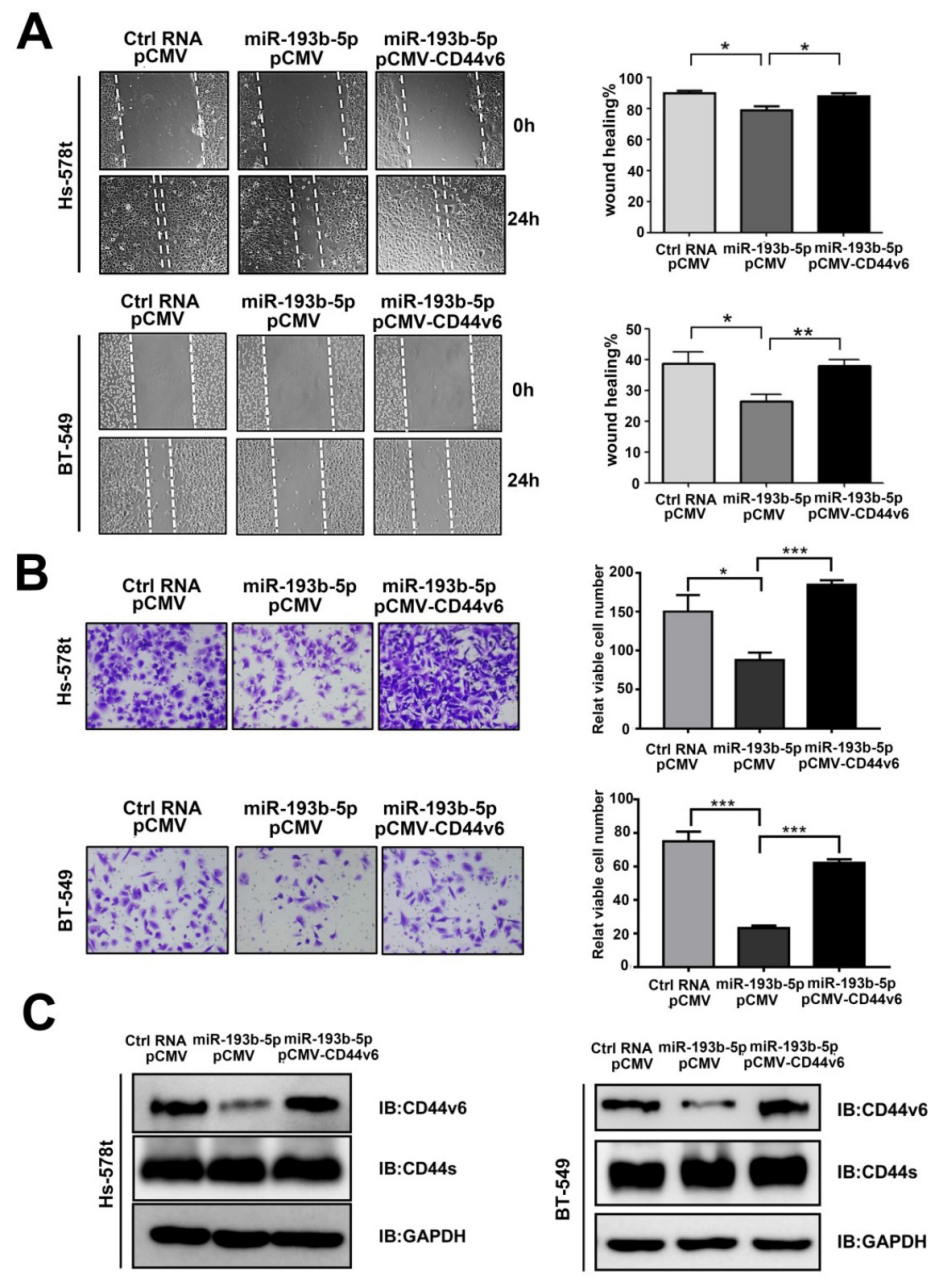
** miR-193b-5p inhibits the migration and invasion of breast cancer cells triggered by CD44v6** (A, B) miR-193b-5p inhibited the migration (A) and invasion (B) of Hs-578t and BT-549 cells, whereas CD44v6 overexpression abolished these changes. Quantitative results of migratory cells are shown on the right,*P<0.05; **P<0.01; ***P <0.001. (C) Protein levels of CD44v6 and CD44s in Hs-578t (left) and BT-549 (right) cells overexpressing miR-193b-5p mimic and CD44v6.

Then, we examined the function of miR-193b-5p in breast cancer aggressiveness. *In vitro* studies showed that miR-193b-5p overexpression in Hs-578t and BT-549 cells significantly reduced cell migration in wound healing assays (Figure 3A) and invasion in transwell Matrigel cell assays (Figure 3B). To observe whether miR-193b-5p played a role in inhibiting migration and invasion by targeting CD44v6, Hs-578t and BT-549 cells were cotransfected with Ctrl-RNA or miR-193b-5p mimics together with an empty vector or the CD44v6 vector. The rescue experiments showed that the addition of the CD44v6 vector reversed the inhibitory status caused by miR-193b-5p (Figure 3A-C). Collectively, these results suggest that miR-193b-5p can inhibit the migration and metastasis of breast cancer cells triggered by CD44v6, and that the restoration of CD44v6 rescues the effects of miR-193b-5p in breast cancer cell lines.

### miR-193b-5p has no effect on the proliferation of breast cancer cells

**Figure 4:**
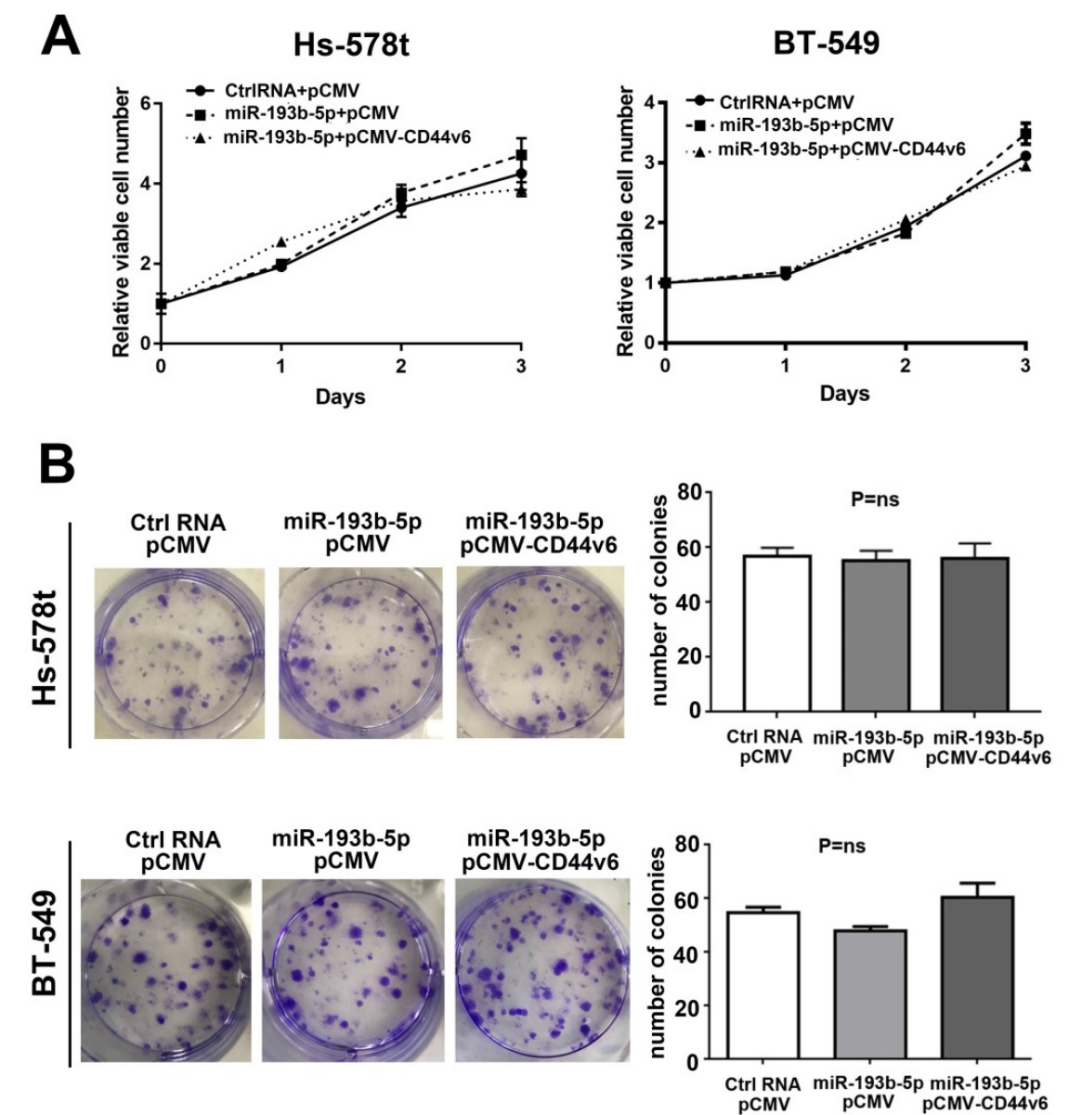
** miR-193b-5p had no effect on the proliferation of breast cancer cells** (A) CCK-8 proliferation assay for vector-, miR-193b-5p mimic- and CD44v6-transfected cells. No obvious difference was observed, P=ns.(B) Quantification of the colony formation assay and results in Hs-578t and BT-549 cells

According to previous research, CD44v6 can promote colon cancer cell proliferation^29^ but has no effect on head and neck squamous cell carcinoma 30. To investigate the effect of miR-193b-5p and CD44v6 on the proliferation of breast cancer cells, we performed CCK-8 and colony formation assays after the cotransfection of Ctrl-RNA or miR-193b-5p with the CD44v6 vector in Hs-578t and BT-549 cells. As shown in Figure 4A, miR-193b-5p barely altered cell proliferation, even with CD44v6 restoration, compared with the controls. The colony assay revealed the same results (Figure 4B). Collectively, our data demonstrate that miR-193b-5p does not affect breast cancer cell proliferation.

### Serum miR-193b-5p is a potential biomarker for breast cancer diagnosis

**Figure 5:**
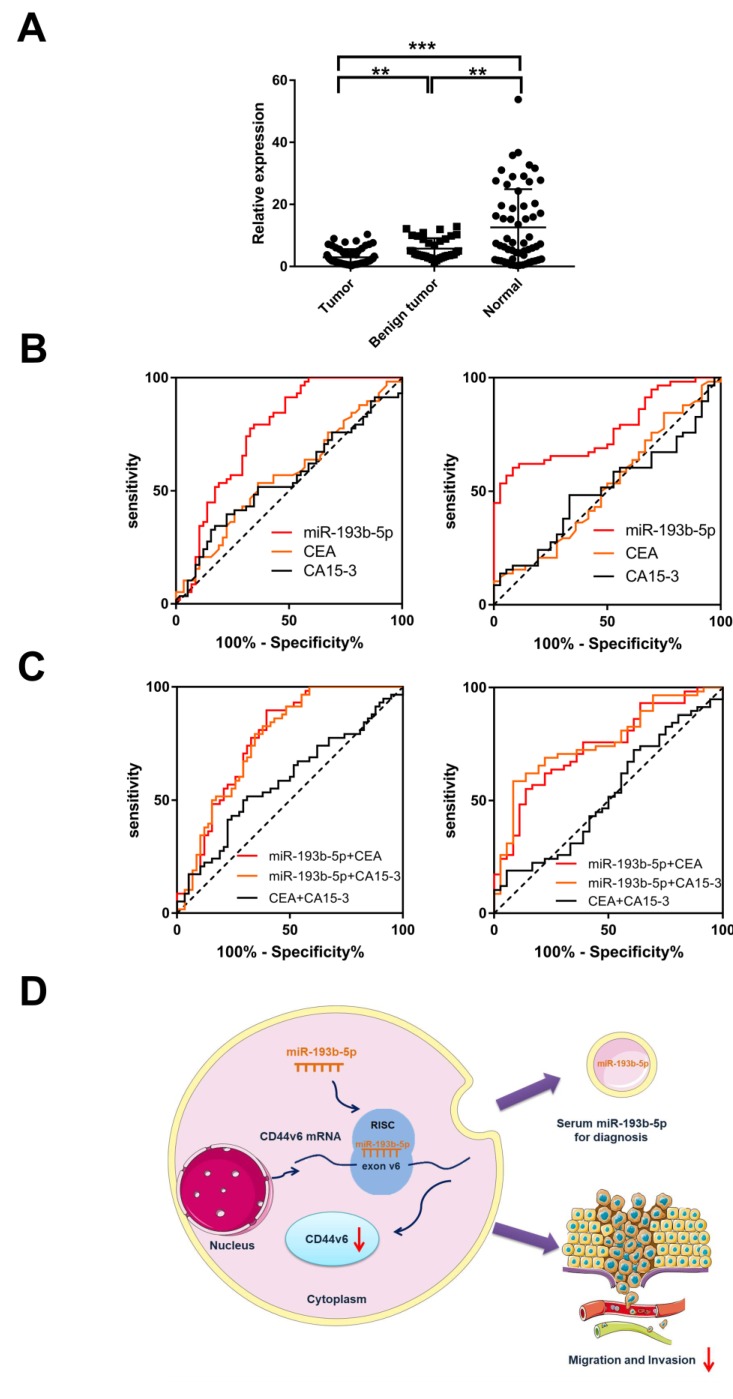
** Identification of miR-193b-5p in the serum of breast cancer patients and comparison with those with benign disease and healthy controls** (A) Scatterplots of the relative expression levels of miR-193b-5p in the serum from healthy controls, benign tumor patients and breast cancer patients. miR-193b5p expression was calculated and is expressed as the miR-193b-5p/U6 expression ratio. (B) ROC curve analysis comparing breast cancer patients, healthy controls, and benign patients by miR-193b-5p, CEA and CA15-3. Left, ROC analysis comparing breast cancer patients with healthy controls by miR-193b-5p, CEA and CA15-3. Right, ROC analysis comparing breast cancer patients with benign disease patients by miR-193b-5p, CEA and CA15-3. (C) ROC curve analysis to identify breast cancer patients, healthy controls, and benign patients by miR-193b-5p combined with CEA or CA15-3 and CEA combined with CA15-3. Left, ROC analysis comparing breast cancer patients with healthy controls by miR-193b-5p+CEA, miR-193b-5p+CA15-3, and CEA+CA15-3. Right, ROC analysis comparing breast cancer patients with benign disease patients by miR-193b-5p+CEA, miR-193b-5p+CA15-3, and CEA+CA15-3. **P<0.01;***P<0.001. (D) Working model of the miR-193b-5p:CD44v6 regulatory axis in breast cancer.

In this study, we found that miR-193b-5p expression was lower in invasive breast cancer cell lines (Figure S1 B) and inhibited migration and invasion by targeting CD44v6. Based on previous studies showing the potential role of miRNA in peripheral blood as a biomarker, we determined the expression level of miR-193b-5p in the serum of breast cancer patients by qRT-PCR assays. The results showed that miR-193b-5p had the lowest expression level in cancer patients, suggesting that miR-193b-5p could be used to distinguish breast cancer from benign disease and healthy controls (Figure 5A).

ROC analysis was used to evaluate the diagnostic value of miR-193b-5p compared with serum biomarkers CEA and CA15-3 in breast cancer and control samples. The area under the ROC curves (AUCs) for miR-193b-5p was 0.762, which was higher than that of CEA (0.567) and CA15-3 (0.552; Table 2 and Figure 5B). When comparing breast cancer with benign disease, we found that the AUC of miR-193b-5p was 0.770. This result was also higher than that of CEA (0.516) and CA15-3 (0.506; Table 2 and Figure 5B), which were poor discriminators of breast cancer in this cohort. These data demonstrate that miR-193b-5p could serve as a biomarker to discern breast cancer patients from healthy controls and patients with benign disease (Table 1 and Figure 5B). Moreover, we combined miR-193b-5p with CEA or CA15-3 to discriminate breast cancer by using ROC analysis. The AUC for miR-193b-5p with CEA or CA15-3 was 0.773 and 0.767, respectively, which were higher than that of the CEA and CA15-3 combination (0.583) (Table 2 and Figure 5C). When combining miR-193b-5p with CEA or CA15-3, the AUC was 0.738 and 0.760, respectively, which were also higher than that of the CEA and CA15-3 combination (0.525). These results indicate that combining miR-193b-5p with CEA or CA15-3 could improve the diagnostic performance of breast cancer. To determine whether miR-193b-5p was affected by the clinical factors of breast cancer patients, we analyzed the relationship between miR-193b-5p expression and pathological characteristics, but no obvious difference was found (Table 2). Collectively, these results indicate that serum miR-193b-5p may function as a biomarker to distinguish breast cancer patients from healthy controls.

## Discussion

First, our results showed that CD44v6-regulated migration and invasion could be inhibited by miR-193b-5p *in vitro*. We found that miR-193b-5p in the serum could function as a biomarker for breast cancer (Figure 5D). Although the expression of CD44v6 correlated with tumor invasion and metastasis 31, the molecular mechanisms involved are poorly understood. In this study, we found that miR-193b-5p is a potential target to CD44v6.

The upregulation of miR-193b-5p inhibited CD44v6 expression, resulting in decreased cell migration and invasion. Moreover, the differential expression of serum miR-193b-5p in patients and cancer-free controls could contribute to breast cancer diagnosis.

Accumulative evidence indicates that CD44v6 is necessary for breast cancer metastasis. Its ample isoforms have been associated with cancer progression 32. CD44v6 overexpression has been found in various cancer types and contributes to tumor progression based on basic and clinical studies 33. Recent studies found that CD44v6 was highly expressed in many squamous cell carcinomas and adenocarcinomas, and its positive rate correlated with tumor metastasis 34-36. The aberrant expression of CD44v6 in other cancers prompted us to determine its expression status and function in breast cancer. In the present study, we found that CD44v6 expression was relatively high in the invasive cell lines Hs-578t and BT-549 compared with the low invasive cell lines MCF-7 and T-47D. The knockdown of CD44v6 in Hs-578t and BT-549 cells demonstrated that CD44v6 possessed the ability to promote tumor migration and invasion. CD44v6 overexpression and function in invasive breast cancer cells collectively suggest that it plays an important role in breast cancer metastasis and may serve as a therapeutic target for breast cancer.

miRNA regulation provides a new research angle for us to study CD44v6. The expression of CD44v6 can promote functional changes in tumor cell surface adhesion to a certain extent, thus promoting the metastasis ability of tumor cells. CD44v6 highly expressed cancer cells can escape killing and recognition by the human immune system and obtain entry into the lymph nodes, thereby forming metastases 37. To examine the significant role of CD44v6, many targeted antibodies were generated to be used in research 34. However, because of the large size of these antibodies, it is extremely difficult to penetrate tumor cells and they have dramatic side effects on the human body 38. It is important to treat this problem from a unique angle. The characteristics of miRNA provided us with new ideas. Data from several studies indicated that miRNAs function as regulators in tumor metastasis by binding to their target genes in the untranslated region(UTR) 39 or in coding regions 40. Because numerous CD44 isoforms have relatively conserved 3'-UTR, it was difficult to determine miRNA function on specific CD44 isoforms. The only difference between CD44v6 and CD44s is the exon v6 41. In this study, miRNAs that bind to the exon v6 were specifically screened and miR-193b-5p was identified as a potential miRNA.

The miR-193b family, which includes miR-193b-3p and miR-193b-5p, is active in tumor progression. According to published data, miR-193b-3p has been widely explored as a tumor suppressor in different cancers such as breast cancer 42, ovarian cancer 43, and acute myeloid leukemia 44. miR-193b-5p together with miR-193b-3p was repressed in dedifferentiated liposarcoma accompanied 45. miR-193b-5p could induce apoptosis and decrease clonogenicity by targeting Polo-like kinase1(PLK1) 46. In this study, miR-193b-5p directly targeted CD44v6 in exon v6 region and was expressed at lower levels in the invasive cell lines Hs-578t and BT-549. These results provide new evidence that miRNAs could target special genes in the coding sequence (CDS). Importantly, functional experiments showed that miR-193b-5p can inhibit the migration and invasion of breast cancer cells by targeting CD44v6 but not CD44s, which proved our point at the protein level. CD44v6 plays a critical role in cancer metastasis, but its role in proliferation remains unclear. CD44v6 can promote cell proliferation in various cancers except for head and neck squamous cell carcinoma 28 and in SW480 cell 14. In this study, we found that CD44v6 had no influence on cell proliferation targeted by miR-193b-5p. In conclusion, our findings indicated that the miR-193b-5p:CD44v6 axis is necessary for breast cancer metastasis and it provide us a new way to explore CD44v6 regulation.

miR-193b-5p in the serum may be a new biomarker for breast cancer. miRNAs affect the expression of many functional genes through posttranscriptional regulation, which is closely related to the occurrence and development of tumors 47. Although there are many experimental results demonstrating that miRNAs have an important influence on the function of tumor development, miRNAs in tissues still have significant limitations as biomarkers. Since the expression of miRNAs in the serum and plasma has proven stable 48, circulating miRNAs may act as potential biomarkers for tumor diagnosis and therapy 49-51. Recent studies have shown that miRNAs in the serum could discriminate pancreatic cancer patients from controls 52. The expression of miRNAs can be detected by RT-PCR and microarray analysis 48, 52. Then, miR-193b-5p expression in the serum from cancer patients and controls was detected. The expression profile obtained by qRT-PCR showed that miR-193b-5p expression was higher in healthy controls than in patients with benign disease and in those with breast cancer. CEA and CA15-3 have been used as breast cancer biomarkers, but they lack sensitivity to the diagnosis of breast cancer 53. In our study, the AUC of miR-193b-5p was higher than that of CEA and CA15-3. CEA and CA15-3 showed low sensitivity in detecting breast cancer, consistent with previous research. In addition, combining miR-193b-5p with CEA or CA15-3 resulted in a higher AUC than the combination of CEA and CA15-3, suggesting that this serum biomarker combination could improve diagnostic values. Then, we analyzed the relationship between miR-193b-5p expression and the clinical factors of breast cancer patients, but no obvious difference was observed. Because of the low expression of miR-193b-5p in breast cancer patients, it might be hard to find obvious differences. The other reason may be that the sample size included in this study was relatively small. Therefore, more samples are required to confirm our results in further studies.

In summary, we discovered that miR-193b-5p could suppress the migration and invasion of breast cancer cells by directly targeting CD44v6 in exon v6. Furthermore, we identified that miR-193b-5p in the serum could discern breast cancer patients from those with benign disease and healthy controls, serving as a potential biomarker for breast cancer diagnosis. The physiological functions of miR-193b-5p with CD44v6 will be further explored.

## Supplementary Material

Supplementary figures and tables.

## Figures and Tables

**Table 1:** Correlation between the levels of miR-193b-5p expression and clinicopathological characteristics

Clinicopathologic parameter	N	miR-193b-5p expression	Median	95% CI	P-value
**Age**					
<50 yr	26	2.847±0.491	1.643	1.837-3.858	0.712
≥50 yr	32	3.106±0.485	1.800	2.116-4.095	
**Histologic type**					
Invasive duct	49	3.105±0.374	2.029	2.353-3.856	0.441
Other	9	2.365±0.897	1.219	0.295-4.434	
**Tumor Size**					
<2 cm	27	2.986±0.452	1.831	2.058-3.915	0.993
≥2 cm	31	2.993±0.516	1.767	1.939-4.047	
**T stage**					
T1	23	3.656±0.632	2.029	2.345-4.966	0.246
T2	28	2.687±0.433	1.710	1.798-3.576	
T3	7	2.013±0.808	0.941	0.036-3.989	
**N stage**					
N0	35	2.817±0.470	1.504	1.862-3.773	0.541
N1/2/3	23	3.252±0.498	3.019	2.220-4.284	
**Histologic grade**					
I	4	1.205±0.079	1.192	1.080-1.330	0.262
II	31	2.862±0.466	1.767	1.910-3.813	
III	23	3.472±0.580	2.554	2.269-4.676	
Ki-67					
<20%	21	3.690±0.588	3.305	2.464-4.916	0.126
≥20%	37	2.592±0.415	1.520	1.750-3.434	
**ER**					
Negative	15	2.941±0.800	1.407	1.226-4.656	0.934
Positive	43	3.007±0.377	1.831	2.246-3.767	
**PR**					
Negative	20	2.883±0.690	1.387	1.438-4.328	0.824
**Positive**	38	3.046±0.386	1.996	2.264-3.828	
HER2					
Negative	25	2.398±0.451	1.504	1.466-3.329	0.135
Positive	33	3.438±0.490	2.465	2.441-4.436	

ER: Estrogen receptor, PR: Progesterone receptor, HER2: Human epidermal factor 2.

**Table 2:** Diagnostic values of miR-193b-5p, CEA, and CA15-3 for breast cancer.

Markers	AUC	95% CI	Sensitivity		
			90% specificity	95% CI	Cut off
Breast cancer versus normal control					
miR-193b-5p	0.762^***^	0.674-0.851	34.48%	0.225-0.481	1.23
CEA	0.567	0.463-0.672	15.52%	0.073-0.274	3.15
CA15-3	0.552	0.447-0.658	17.91%	0.050-0.233	17.91
miR-193b-5p+CEA	0.773^***^	0.687-0.860	25.86%	0.153-0.391	
miR-193b-5p+CA15-3	0.767^***^	0.680-0.854	27.59%	0.167-0.409	
CEA+CA15-3	0.583	0.489-0.688	17.24%	0.086-0.294	
					
Breast cancer versus benign disease					
miR-193b-5p	0.770^***^	0.677-0.863	60.34%	0.466-0.730	2.49
CEA	0.516	0.395-0.638	11.11%	0.031-0.261	0.86
CA15-3	0.506	0.388-0.624	16.17%	0.086-0.294	16.2
miR-193b-5p+CEA	0.738^**^	0.638-0.840	34.48%	0.225-0.482	
miR-193b-5p+CA15-3	0.760^***^	0.662-0.859	58.62%	0.449-0.714	
CEA+CA15-3	0.525	0.404-0.646	18.97%	0.099-0.314	

**P<0.01; ***P <0.001
