# Supplementary material for: CD44v6 Targeted by miR-193b-5p in the Coding Region Modulates the Migration and Invasion of Breast Cancer Cells
Source: J Cancer. 2020 Jan 1;11(1):260–71. doi: 10.7150/jca.35067 (PMC6930394; doi:10.7150/jca.35067)
Supplement: Supplementary file 1 — Supplementary figures and tables. [file jcav11p0260s1.pdf]

Figure S1

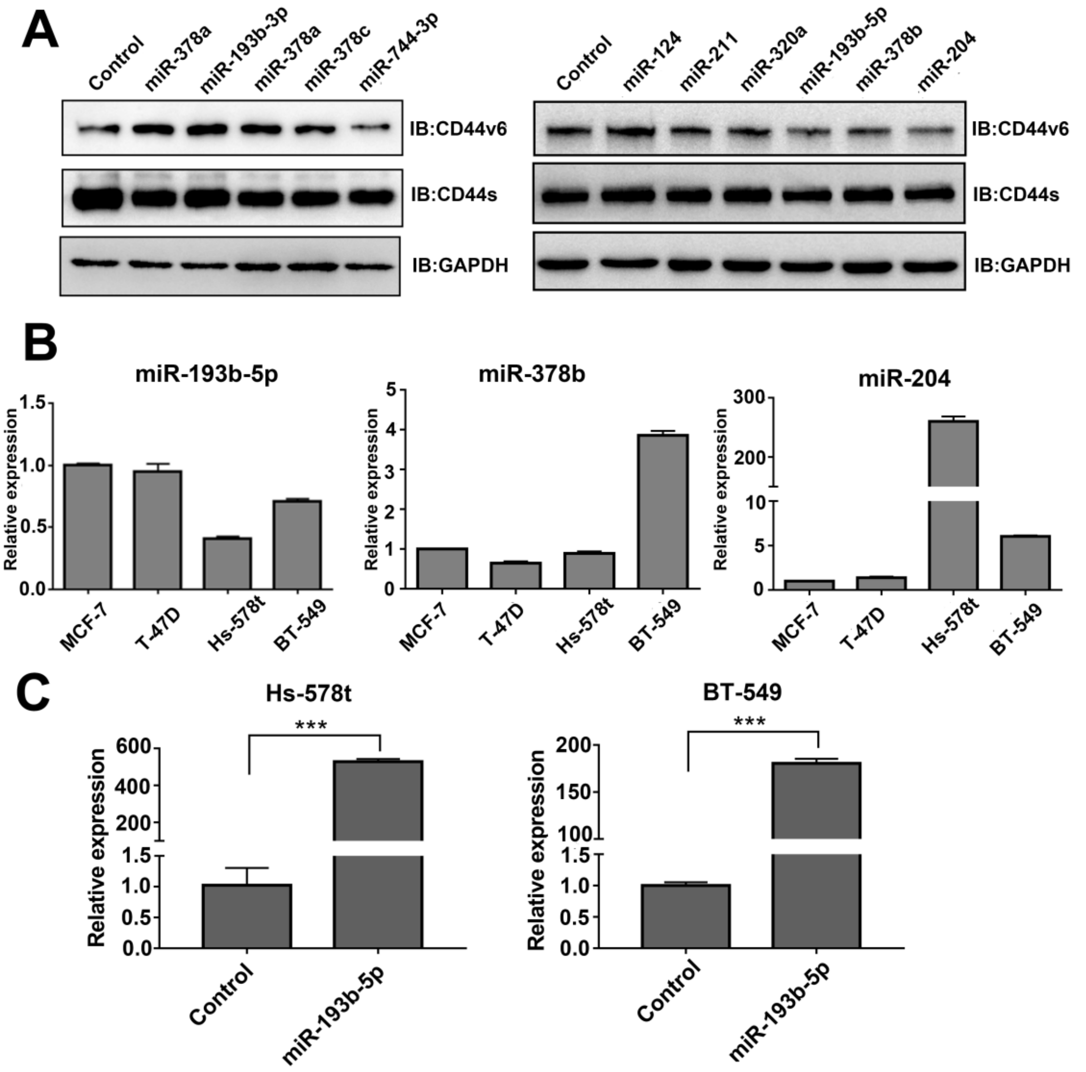

Figure S1 miR-193b-5p, miR-378b and miR-204 may target CD44v6

(A) Western blot analysis of CD44v6 expression in BT-549 cells transfected with 11 miRNAs. (B) qRT-PCR analysis of miR-193b-5p, miR-378b and miR-204 in the breast cancer cell lines MCF-7, T-47D, Hs-578t and BT-549. (C) qRT-PCR analysis of miR-193b-5p after the miRNA transfection in Hs-578t and BT-549. ***P<0.01

Table S1 Sequences of chemically synthesized oligonucleotides

	CD44v6	The forward primer<br>5'-TGCTACCATCCAGGCAACT-3'<br>The reverse primer<br>5'-ATCATTCCTATCAGCTGTCC-3'
	CD44-exon v6	The forward primer<br>5'-TGCTCTAGATCCAGGCAACTCCTAGTAGT-3'<br>The reverse primer<br>5'-AAGGAAAAAAGCGGCCGCCAGCTGTCCCTGTTG<br>TCGAA-3'
Primers for CD44v6 and GAPDH	GAPDH	The forward primer<br>5'-AGCCTCAAGATCATCAGC-3'<br>The reverse primer<br>5'-GAGTCCTTCCACGATACC-3'
	CD44 exon v6-Mut1	The forward primer<br>5'-TGCTCTAGAGTAGTACAACGGAAGAAACA-3'<br>The reverse primer<br>5'-AAGGAAAAAAGCGGCCGCCAGCTGTCCCTGTTG<br>TCGAA-3'
	CD44 exon v6-Mut2	The forward primer<br>5'-TAATTCTAGATCCAGGCAACGGATAGTAGTACA<br>ACGGAAGAA-3'<br>The reverse primer<br>5'-TTCTTCCGTTGTACTACTATCCGTTGCCTGGATC<br>TAGAATTA-3'
	miR-193b-5p	miR-193b-5p-RT<br>5'-CTCAACTGGTGTCGTGGAGTCGGCAATTCAGTT<br>GAGTCATCTCG-3'<br>The forward primer<br>5'-ACACTCCAGCTGGGCGGGGTTTTGAGGGCG-3'<br>The reverse primer<br>5'-CTCAACTGGTGTCGTGGAGTCGG -3'
Primers for miRNAs	miR-378b	miR-378b-RT<br>5'-CTCAACTGGTGTCGTGGAGTCGGCAATTCAGTT<br>GAGTTCTGCCT-3'<br>The forward primer<br>5'-ACACTCCAGCTGGGACTGGACTTGGAGGCA-3'<br>The reverse primer<br>5'-CTCAACTGGTGTCGTGGAGTCGG -3'
	miR-204	miR-204-RT<br>5'-TCAACTGGTGTCGTGGAGTCGGCAATTCAGTTG<br>AGAGGCATAG-3'<br>The forward primer

		5'-ACACTCCAGCTGGGTTCCCTTTGTCATCCT-3'<br>The reverse primer<br>5'-CTCAACTGGTGTCGTGGAGTCGG -3'
	U6	U6-RT<br>5'-CTCAACTGGTGTCGTGGAGTCGGCAATTCAGTTGAGAAAATATG-3'<br>The forward primer<br>5'-ACACTCCAGCTGGGCGCAAATTCGTGAAGC-3'<br>The reverse primer<br>5'-CTCAACTGGTGTCGTGGAGTCGG -3'
siRNA and miRNA	CD44v6 siRNA<br>control siRNA<br>miR-193b-5p mimic<br>control mimic	5'-CAGUGGUUUGGCAACAGAUTT-3'<br>5'-GCGACGAUCUGCCUAAGA-3'<br>5'-CGGGGUUUUGAGGGCGAGAUGA-3'<br>5'-UUUGUACUACACAAAAGUACUG-3'
